# Optimizing the process of fertility preservation in pediatric female cancer patients – a multidisciplinary program

**DOI:** 10.1186/s12885-016-2584-7

**Published:** 2016-08-09

**Authors:** Irit Ben-Aharon, R. Abir, G. Perl, J. Stein, G. Gilad, H. Toledano, S. Elitzur, G. Avrahami, A. Ben-Haroush, G. Oron, E. Freud, D. Kravarusic, M. Ben-Arush, G. Herzel, I. Yaniv, S. M. Stemmer, B. Fisch, S. Ash

**Affiliations:** 1 Institute of Oncology, Davidoff Center, Rabin Medical Center Petah Tikvah and Sackler Faculty of Medicine, Tel Aviv University, Tel Aviv, Israel; 2 IVF and Infertility Unit, Schneider Women Hospital, Rabin Medical Center Petah Tikvah and Sackler Faculty of Medicine, Tel Aviv University, Tel Aviv, Israel; 3 Department of Pediatric Oncology, Schneider Children’s Hospital, Petah Tikvah and Sackler Faculty of Medicine, Tel Aviv University, Tel Aviv, Israel; 4 Department of Pediatric Surgery, Schneider Children’s Hospital, Petah Tikvah and Sackler Faculty of Medicine, Tel Aviv University, Tel Aviv, Israel; 5 Division of Pediatric Hematology Oncology, Ruth Rappaport Children’s Hospital, Rambam Health Care Campus, Haifa, Israel; 6 Department of Pediatric Hematology Oncology, Ha’Emek Hospital, Afula, Israel

**Keywords:** Pediatric cancer patients, Fertility preservation, Ovarian cryopreservation

## Abstract

**Background:**

Current evidence indicates sub-optimal incidence of fertility preservation (FP) in eligible patients. We present herein our designated multidisciplinary program for FP in pediatric and adolescent population and present our data on FP in female patients.

**Methods:**

Pediatric patients (age 0–18) who were candidate for highly gonadotoxic treatments were referred to FP program for a multidisciplinary discussion and gonadal risk-assessment followed by either oocyte cryopreservation or ovarian cryopreservation (OCP) for female patients, and sperm banking for male patients. The OCP protocol consists of aspiration of oocytes from small antral follicles and in-vitro maturation followed by cryopreservation, as well as ovarian tissue cryopreservation.

**Results:**

The establishment of a designated FP program resulted in a significant increase in referral and subsequent FP procedures of all eligible patients. Sixty-two female patients were referred for FP discussion during a period of 36 months; 41 underwent OCP; 11 underwent oocyte cryopreservation and six were declined due to parental decision. The median age was 13.2y (range 18 months-18y). Thirty-two (51.6 %) were chemotherapy-naïve. Seventeen patients (27 %) had sarcoma, 16 patients (26 %) had acute leukemia. The mean number of mature oocytes that were eventually vitrified was significantly higher in chemotherapy-naïve patients compared with chemotherapy-exposed patients (mean 12 oocytes (1–42) versus 2 (0–7)).

**Conclusion:**

Multidisciplinary programs that encompass experts of all relevant fields, skilled laboratory resources and a facilitated path appear to maximize the yield. We observed a considerable higher referral rates following launching a designated program and earlier OCP in chemo-naïve patients that culminated in a better fertility preservation procedure.

## Background

Approximately 12,000 adolescents and children under the age of 18 are diagnosed with cancer each year in the United States. Fortunately, therapeutic advances have resulted in increased survival while five-year survival rate for pediatric cancer currently approaches 80 % [1, 2]. Cancer during childhood and adolescence introduces major challenges regarding treatment-related late effects, and as survival rate improves, there is much attention on interventions to improve the quality of life during survivorship [2].

Up until 2013, the standard methods to preserve fertility were ovarian transposition for female patients undergoing pelvic radiation and embryo cryopreservation for pubertal female patients before initiation of gonadotoxic chemotherapy, whereas oocyte preservation and ovarian cryopreservation have been considered experimental. Recently, the American Society for Reproductive Medicine (ASRM) revised its guidelines for fertility preservation (FP) in cancer patients and acknowledged oocyte harvesting and cryopreservation as an ancillary standard procedure of FP prior to gonadotoxic treatment. Following the establishment of ASRM that pregnancy rates resemble in both oocyte cryopreservation and embryo cryopreservation; the American Society of Clinical Oncology (ASCO) has concurred its recent guidelines with those of the ASRM, stating oocyte cryopreservation is recommended as a standard option for FP for pubertal females [3]. This revision carries highest impact for young pubertal female patients that until recently were asked to preserve embryos using donor sperm, with complicated future implications who now may undergo a much useful methodology that may better serve their future needs whether facing late-term infertility. For prepubertal girls, ovarian tissue cryopreservation is the only option to restore gametes. Since the benefit to the patient is not yet established, ASCO and ASRM guidelines state that ovarian tissue cryopreservation should be considered experimental and offered only in a research setting with Institutional Review Board (IRB) approval [3]. Ovarian cryopreservation has been widely studied and methods have improved in many cancer centers worldwide [4–6].

Fertility preservation in pediatric and adolescent patients has gained better appreciation throughout the past years with improving rates of ovarian cryopreservation and continuous crosstalk and collaborative frameworks between oncologists and reproductive endocrinologists and with the advent of skilled laboratories that handle ovarian tissue [3, 4, 6].

This unique population presents psychosocial challenges derived from the decisions about FP, which may have a substantial impact on the adolescent as an adult survivor. For the pediatric patient, these bothersome decisions are taken by her caregiver, what may aggravate the primary stressful situation [7].

Former studies indicate that adolescents with cancer appreciate information prior to initiation of treatment on gonadal toxicity and potential impact to future fertility, valid options for FP and wish to take active part in the decision making process regarding FP [7]. Several studies confirm that adult survivors of pediatric cancer regret they had not been given more detailed information of options about FP, even though experimental [8–10]. For parents feeling uncertain in making the decision about FP on behalf of their young girl, the ASRM [8] offers guidance for counseling parents of children with cancer of either sperm banking or ovarian cryopreservation. Several studies have implied that establishing the right setting for fertility counseling for young patients and parents regarding fertility and cancer treatments carries high implications to alleviate the psychological stressors associated with both the acknowledgement of cancer diagnosis and the risk of future infertility [9–11].

Fertility represents a key issue in cancer survivorship of pediatric or young adult patients. Follow-up surveys developed by ASCO aiming at cancer survivors indicate that those who are infertile following anti-cancer treatments are at increased risk for emotional distress [12–14]. Yet, despite the paramount significance, the current evidence points at a sub-optimal approach to discussion of FP by the health care providers reflected by still lower referral numbers of eligible patients to FP or even routine discussion on this matter. The literature implies that although most oncologists recognize the importance of raising the issue of treatment-related infertility, the actual lack of discussion may derive from desire to commence immediate treatment and sub optimal connection between oncologists and reproductive endocrinologists [11–13].

On this ground we have developed a unique program that aimed at facilitating the daily practice and collaboration between the various contributors to the process of fertility preservation, emphasizing practical issues of enhancing the end-products of the process, focusing on the psychosocial aspects and utilizing this platform to prospectively assess gonadotoxicity throughout time following anti-cancer treatments under IRB approval. The program serves male patients (for sperm banking and recently a newly approved protocol of testicular biopsy cryopreservation) and female patients (oocyte and ovarian cryopreservation). The availability of providing banking of gonadal tissue requires a unified multidisciplinary team with appropriate laboratory resources. We herein present our paradigm, the shift in referral of pediatric and adolescent patients in the last 36 months following launching the program and our experience of fertility preservation in prepubertal and post-pubertal young female cancer patients.

## Methods

### Patients

Pediatric patients (0 – 18 years) from three cancer centers (Schneider Children’s Medical Center/Rabin Medical Center (SCMC/RMC), Ruth Rappaport Children's Hospital, Rambam Health Care Campus, Haifa and Ha’emeck Medical Center in Afula, Israel) who were candidates for highly or intermediate-high gonadotoxic treatment (i.e., alkylating agent-based, induction chemotherapy for bone marrow transplantation (BMT), pelvic/abdomen radiation or high-dose platinum based protocol) were referred to the program. Following a referral algorithm (see below), and in the presence of psychosocial counselor, the parents (and also the patient, in case of adolescents (>12 years)) signed the informed consent for FP procedure as well as an informed consent for the longitudinal follow up on gonadotoxicity (the protocol was approved by the institutional review board of Rabin Medical Center/Schneider Children’s Medical Center, to which all patients were referred; RMC −11-6549). Prepubertal girls were referred for ovarian tissue cryopreservation while pubertal girls were referred for oocyte cryopreservation if possible according to time constraints defined by her oncologist and time during menstrual cycle/first day of menstruation. In case of highly gonadotoxic regimen administered to pubertal girls that have not been exposed recently to chemotherapy, the goal was to have both ovulation induction and oocyte cryopreservation and afterwards ovarian tissue cryopreservation due to the very high probability for gonadal failure after treatment.

On the first appointment a questionnaire containing demographic and medical obstetrical information is being collected. Before fertility preservation – either ovulation induction or ovarian retrieval, blood is drawn for hormonal profile (Follicle Stimulating Hormone (FSH), Luteinizing Hormone (LH), Estradiol) and Anti Mullerian Hormone (AMH). In case of ovarian cryopreservation, a second tube of blood is drawn after surgery to set a baseline value with less ovarian tissue and before the initiation of chemotherapy. Serum is being drawn every 6 months in the first year and annually from the second year for the same factors.

### Fertility preservation options in pediatric and adolescent female patients

**Fig. 1:**
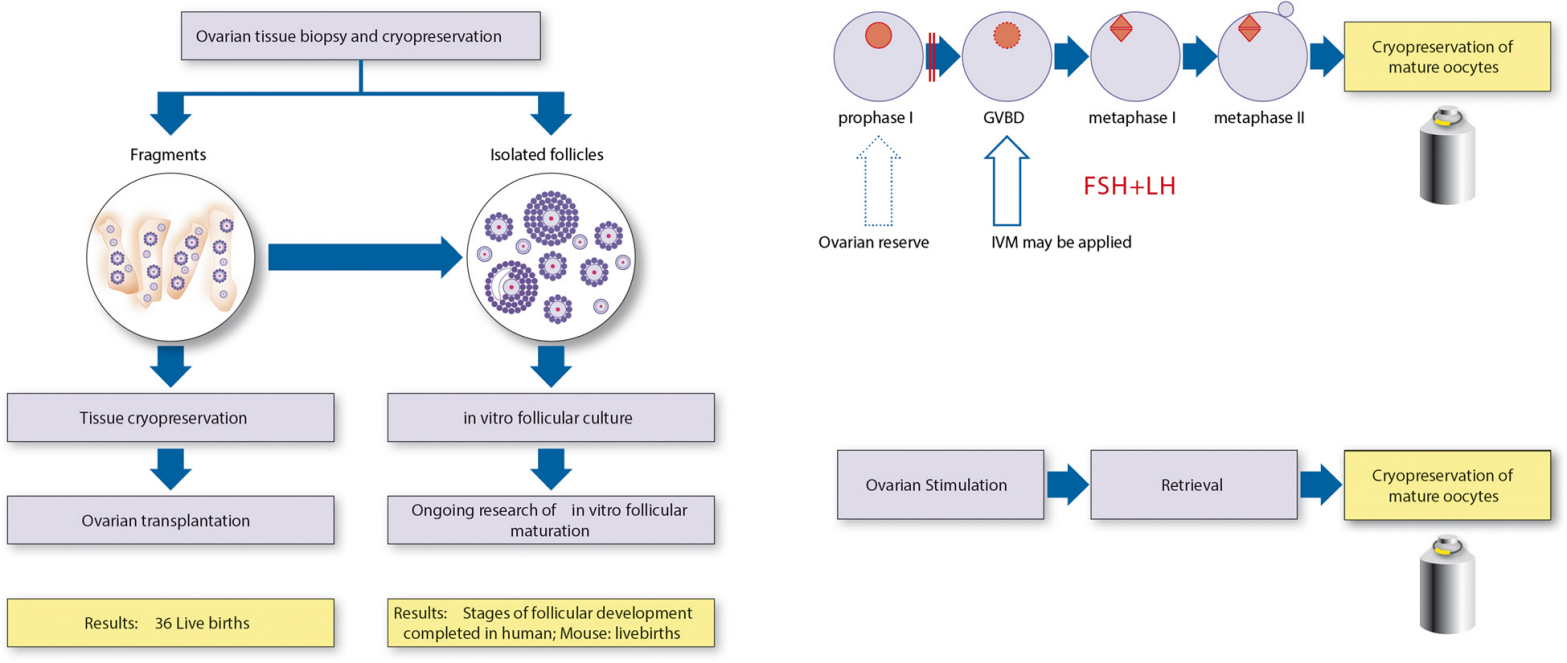
FP options for prepubertal girls and pubertal adolescents. **a** Current evidence for ovarian cryopreservation. **b** Schematic representation of in vitro oocyte maturation (IVM) and subsequent cryopreservation. **c** Ovulation induction and oocyte retrieval and cryopreservation

Figure 1 illustrates the options for fertility preservation in young female patients. For prepubertal patients ovarian tissue cryopreservation is considered the only valid option, yet it is considered experimental and requires IRB approval. This procedure entails removal of tissue from part or all of an ovary (by the discretion of the surgeon upon the size of the ovary; in very young girls usually a whole ovary is excised) which is then cryopreserved for future use. The future use of the tissue carries two alternatives: reimplanting an ovarian section or the entire ovary into the patient. Several dozens of live births were documented using this technology, yet only in adult patients at the time of ovarian cryopreservation. Ovarian cryopreservation in younger children has been frequently reported, nevertheless, there have been no reports of live births after performing this procedure during prepuberty mainly due to the young age of the treated patients and the relatively short time that had passed since ovarian cryopreservation [15, 16]. National Comprehensive Cancer Network (NCCN) guidelines consider ovarian cryopreservation is an investigational method of FP since the possibility of reseeding cancer cells through reimplanted tissue is still valid [17]. The second use of ovarian tissue is in vitro maturation of primordial follicles. The ovary, especially of young girls is enriched with copious number of primordial follicles that may potentially serve as “oocytes bank” for use. The process of in vitro follicular maturation is being extensively investigated, yet complete maturation of primordial follicles has not yet been achieved yet in human. Results in animal studies are promising due to major advances in recent years [18].

In 2013, ASCO has recognized oocyte harvesting and cryopreservation as a standard option of FP [3]. The full ovulation induction starts at the beginning of menstrual cycle and hence ranges in the required time depending upon the last menses of pubertal patients. In vitro maturation (IVM) of pre-antral follicles enables maturing of immature (growing) follicles in the laboratory and vitrification of mature oocytes. Yet, IVM is not feasible after natural ovulation has occurred, but serve as an axillary option when time constraints do not allow full ovulation induction, while the patient is in the proliferating phase of the menstrual cycle (before ovulation).

### Referral algorithm

**Fig. 2:**
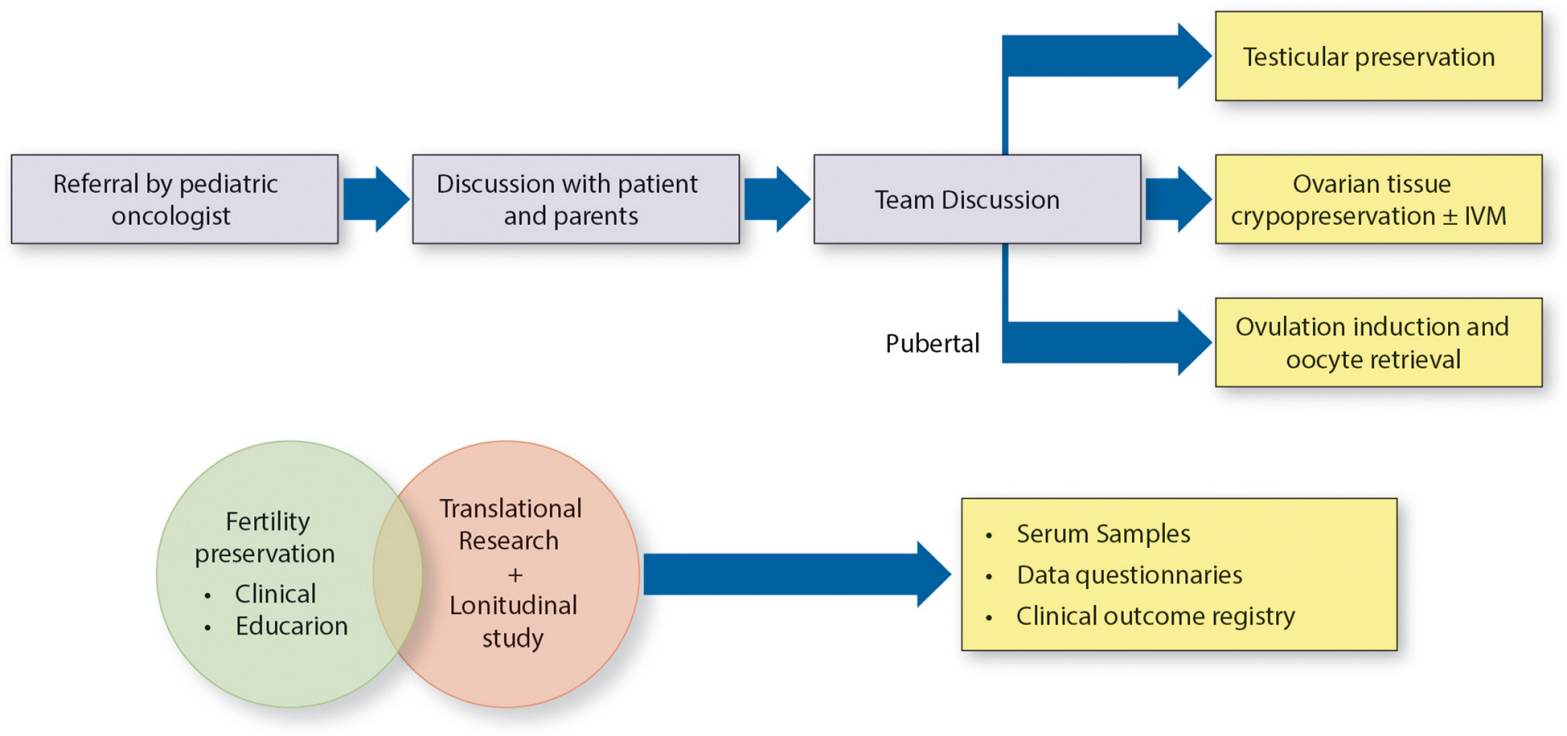
referral algorithm Oncofertility program at RMC. Team is comprised by REI, Oncologist (moderator), Psychologist. The lower part represents the components of the prospective study for longitudinal evaluation of reproductive outcomes in this cohort

Since the establishment of the program, we had refined our multidisciplinary approach upon the patient/parents’ feedback. The program comprised of two parts – the adult program, for young adult female and male patients treated at Davidoff Center, and the pediatric part which during the time had expanded to referral from 3 pediatric cancer centers. The moderator is an oncologist with prior academic background in reproductive biology that is committed to this program and receives a notification from the pediatric oncologist regarding a new patient, the diagnosis, treatment plan and timing (Fig. 2). The first appointment occurs at the cancer center with a detailed discussion, using educational tools about the impact of the treatments on fertility and description of FP options. Usually, the parents are very distressed throughout this session and worry also about the impact of FP on cancer treatment. After this discussion if the parents/adolescent patients express their wish to continue with FP they are referred to a second professional discussion in the reproductive endocrinology facility, by a team consisting of the oncologist (moderator), a reproductive specialist and psychologist who is an integral part of the reproductive endocrinology unit. During this meeting the parents are also asked to sign the informed consent. After this appointment the patient continues for a preoperative assessment. For ovulation induction in pubertal girls there is a continuous consultation with the pediatric oncologist to fit time constraints. In case the patient needs to commence treatment very soon, if she is in the follicular menstrual cycle phase (before ovulation), oocyte aspiration and an in vitro maturation procedure may be performed. Pubertal male patients are referred to sperm banking following the multidisciplinary discussion, and recently we had launched a protocol of testicular biopsy for prepubertal boys and adolescents in whom sperm banking is not feasible.

### Surgical considerations

All patients underwent laparoscopic harvesting of ovarian tissue under general anesthesia using one 10-mm port positioned at the umbilicus and two 5-mm ports, one at the left lower quadrant and one at the right. Partial or complete, unilateral oophorectomy was performed according to the consensus decision of the pediatric oncologist, the surgeon, and the medical staff of the IVF unit, based on ovarian size and the anticancer treatment protocol. The average OR time was 40 min and dissection was carried out avoiding use of any device that may induce collateral electric or thermal injury to the ovarian tissue. Adolescent females with a larger volume of ovarian tissue underwent partial ovariectomy, while pre-pubertal girls with small ovaries underwent complete ovariectomy, according surgeon’s discretion. Ovarian evacuation required an endo-bag, fresh tissue was sampled for pathological examination and the bulk of ovarian tissue was forwarded to the laboratory in a maximum 30 min. No complications were recorded. All patients had a benign postoperative course and were discharged the next day. In follow up, no wound infection or port-side hernias were detected and cosmetic results were excellent. Our preliminary experience with laparoscopic technique was already published [19, 20]

### Ovarian cryopreservation

Prior to ovarian cortex slicing, germinal vesicle (GV)-stage oocyte are being aspirated from small antral follicles (>2 mm) in the operating room or/and manually from the ovarian tissue [21]. Isolated oocytes are incubated in IVM medium for 24 h and all intact oocytes matured 24–48 h post retrieval are being vitrified. Ovarian slices containing primordial follicles are being frozen as well (slow freezing).

### Assessment of referral trends among pediatric oncologists

Female patient database of SCMC was screened and data extracted of all female patients treated with highly gonadotoxic regimens (defined as: high dose alkylating agents, bone marrow transplantation, total body irradiation (TBI)) from 2009–2011 (prior to the establishment of the program) and between 2012–2014 (following launching the program). Referral rates to fertility preservation were assessed by disease for the two corresponding timeframes. Patient data was reviewed for disease type, patient age and prior exposure to chemotherapy.

## Results

### Cohort demographics

**Fig. 3:**
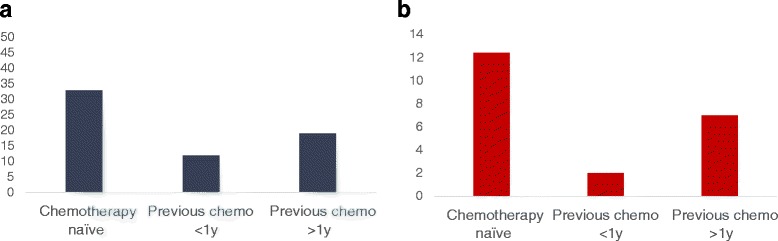
Characteristics of referred patients (**a**) Patient distribution according to cancer type (**b**) Patient distribution according to therapeutic protocol. Abbreviations: HL – Hodgkin’s lymphoma; AML – acute myeloid leukemia; ALL – acute lymphocytic leukemia; STS – soft tissue sarcoma; CNS – central nervous system; BMT – bone marrow transplantation TBI – total body irradiation, XRT – irradiation

The program at Rabin Medical Center/Schneider Children’s Medical Center (same campus) was launched at 1/2012. Prior to the establishment of the program, fertility preservation in pediatric population had been performed in our center, yet not as a designated program but rather on a case to case basis. In 36 months of its activity 62 pediatric patients had been referred from Schneider Children’s Medical Center, Ruth Rappaport Children's Hospital Rambam Health Care Campus, Haifa and Haemeck Medical Center in Afula, Israel, all were referred to the RMC/SCMC program. The median age was 13.2 (range 18 months–18 years). Thirty two (51.6 %) were chemotherapy-naïve. Sixteen patients (26 %) had acute leukemia (ALL and AML), before bone marrow transplantation. Twelve patients were candidates for ovulation induction and oocyte cryopreservation (pubertal patient with regular menses, intermediate-risk protocol and chemotherapy delay was approved by the oncologist). Fifty patients were candidates for ovarian cryopreservation. Of All patients that were referred to FP discussion (n = 62), 41 patients underwent ovarian cryopreservation; 11 underwent oocyte cryopreservation. Six patients withdrew the procedures for parental reasons, and in four cases the planned treatment protocol had been changed to less gonadotoxic protocol and hence FP was declined. Characteristics of referred patients are demonstrated in Fig. 3.

### Reproductive outcomes

**Fig. 4:**
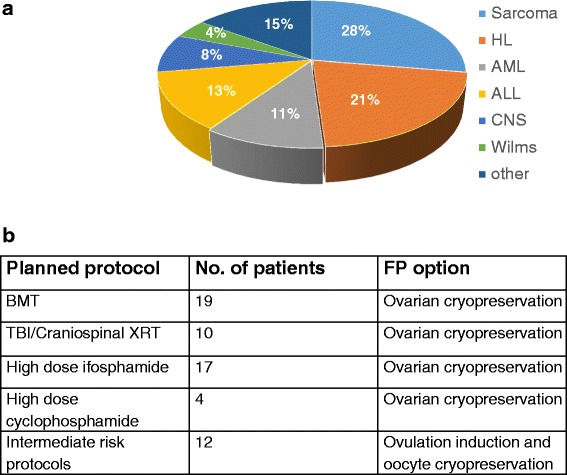
Reproductive outcomes (**a**) Small antral follicles - distribution according to prior chemotherapy exposure (naïve, recent (<1 year), prior chemotherapy (in case of cancer recurrence; >1y)). **b** In Vitro matured (frozen) oocytes; distribution according to prior chemotherapy exposure

Prior to ovarian cortex slicing of the excised tissue exported from the operating room (as described in the method section), germinal vesicle (GV)-stage oocyte were aspirated from small antral follicles (>2 mm) manually from the ovarian tissue. Isolated oocytes were incubated in in vitro maturation (IVM) medium for 24 h and all intact oocytes that matured 24–48 h post retrieval were vitrified. Ovarian slices containing primordial follicles were frozen as well. As presented in Fig. 4, the mean number of mature oocytes that were eventually vitrified was doubled in chemotherapy-naïve patients compared with patients that had previously been treated with chemotherapy (within the last 6 months). For many of the previously-treated patients, no small antral follicles were observed, reflecting the high vulnerability of this follicle population to chemotherapy, even those which is considered low gonadotoxic.

### Shifting paradigms – referral rate before and after the establishment of the program

**Fig. 5:**
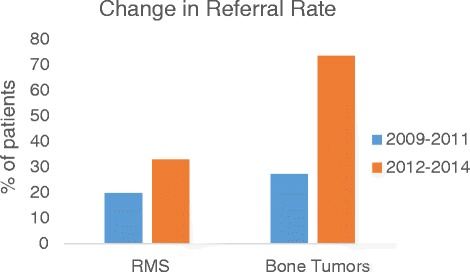
shifting paradigms – referral rate of chemo-naïve patients before and after initiation of a designated program. Bone tumors (Ewing sarcoma and osteosarcoma), RMS - Rhabdomyosarcoma

Schneider Children’s Medical Center of Israel is a tertiary referral center and is considered the largest in Israel. Patient database at SCMC was searched for all female patients eligible for fertility preservation (candidate for highly gonadotoxic regimens) by cancer type and age between 2009–2014. Figure 5 describes the significant change in referral rate in chemotherapy-naïve patient population. Among patients with bone cancer (osteosarcoma and Ewing sarcoma) referral rate increased from 27.3 % to 73.7 % after the establishment of the program (3/11 patients and 11/15 patients respectively). This group also represents mainly post pubertal patients considered for oocyte cryopreservation. Among patients with rhabdomyosarcoma that represent mainly the prepubertal age group, referral rates increased from 20 % in the 3 years before the program was launched to 33 % (1/5 patients and 2/6 patients respectively). Increase in referral rates was documented in other cancer types (brain tumors, lymphoma and high risk leukemia patients who were candidates for BMT). In 2009–2011 most of the patients that were referred to FP were candidates for induction chemotherapy for BMT or high dose alkylating agents, mostly pre-treated patients. Since one of the main rationales of the program was to increase the awareness of the medical team to early referral, the population of chemotherapy-naïve patients represents the major challenge.

Following the initiation of the program, younger patients are being referred after 2012 (6 patients below the age of 10 years and 5 patients below the age of 5 years) as compared to the previous period before 2012 (*t*-test, *p* < 0.05). Data regarding reproductive outcomes (ovarian function, menstruation, future pregnancies) and oncologic outcomes (survival, recurrence etc.) is ongoing prospectively collected.

## Discussion

In an era of improving treatment protocols and subsequent increased survival of pediatric cancer patients, where 5-year survival reaches 80 % [1, 2], fertility preservation has become a pivotal survivorship issue. For younger prepubertal female patients, ovarian tissue cryopreservation carries promise for future use in experimental protocols, such as ovarian transplantation after which live births had occurred, though only while the tissue had been retrieved from adult patients [3]. In vitro follicle maturation has been intensively studied and carries high implication to practice since it is devoid of any risk of disease recrudescence [22–24], especially for survivors of hematologic malignancies.

Former studies have evaluated the adherence of care providers to ASCO recommendation of thorough discussion of fertility and referral to fertility preservation [3]. Studies which assessed the patients’ notion on the issue revealed that most of the adolescent patients as well as parents of pediatric patients felt unsatisfied with the degree of discussion and the very limited information received, and the majority stated that they wished to have a deeper discussion to resolve many unclear questions and thoughts [25–27]. It has been formerly established that other than actual preservation of fertility for the future, the discussion and referral to FP helps the patients to cope better with their condition and treatment. Studies performed in adolescent males who were referred to sperm banking demonstrated that sperm banking was considered by the patients as a strong positive factor in coping emotionally with cancer [27, 28]. A study of testicular preservation in prepubertal boys, revealed that families are willing to undergo an experimental protocol even when there were no guarantees that the science will exist in the future to allow their sons to use this banked tissue to achieve pregnancy [29]. For many parents and adolescents, the discussion of future fertility is acknowledged as a discussion on the “day after” and carries hope [29, 30]. From the medical facet, provider perceptions were appraised in several studies and revealed that although most oncologists recognized the significance of discussing fertility with the patients, in routine practice, less than half of the pediatric oncologists referred the patients to FP [31–33]. In an analysis of the factors that contributed most to the lack of referral to FP, availability of fertility specialist and time constraints, were identified as the most common [31].

Former reports have implied that following development of a designated and standardized process of offering FP to adolescents and young adults, there has been a substantial increase in the proportion of patients that were referred for FP [29, 34–36].

Our comprehensive multidisciplinary program was developed to pursue the complex issues associated with fertility and cancer. We aimed to encompass both fertility preservation form the clinical aspect but also to employ this setting as a platform for translational research on gonadotoxicity. The program was designed to provide care for both male patients (for sperm banking and recently a newly approved protocol of testicular biopsy cryopreservation) and female patients (oocyte and ovarian cryopreservation). From the clinical perspective, our concept lied on the perceptions and needs observed in our patient population on upon the literature. We intended to raise the awareness of the caregivers to the issue of fertility, facilitate the process of referral, and serve as an approachable service for the patient. Once notified by the treating oncologist, an appointment would be scheduled within a short time and the algorithm of referral would be operated. We refined the algorithm of treatment based upon perceptions and feedback from the patients, parents and the pediatric oncologists. We had found that an initial discussion performed in the clinic by a trained oncologist contributed to “priming” of the parents, who were then referred for a broader discussion with the reproductive specialist and a specialized psychologist of the reproductive endocrinology service. Following the algorithm, the parents and adolescents felt less stressful and less concerned of how the FP procedure may affect their cancer (that was mostly evident with adolescent pubertal patients that were undergoing ovulation induction for oocyte cryopreservation). The multidisciplinary paradigm entails a better collaboration between all disciplines – availability of personnel to carry the appointments, flexibility of operation schedule by skilled surgeons, and most importantly appropriate laboratory resources. By meticulous handling of the ovarian tissue, we managed to aspirate immature germinal vesicle oocytes that had already commenced maturation process and to complete the maturation in vitro. By that, the pediatric patient gained additional source of FP and had not only stored ovarian tissue but rather mature oocytes. The multidisciplinary infrastructure enables us to expand our program also to testicular preservation in prepubertal boys, a process which has been implemented very recently. Our multidisciplinary program has led to a significant increase in referrals of pediatric patients. With the advent of oocyte cryopreservation as a standard option of FP, the program facilitated this clinical application reflected by higher referral rates of pubertal patients (that up until recently were referred for embryo cryopreservation with sperm donation, an option that is considered highly problematic for young adolescents). From the research perspective, every patient that is referred to the clinic is enrolled into an IRB-approved prospective longitudinal study of gonadotoxicity related outcomes in young patients who are treated with various anti-cancer therapies. Gonadotoxic outcomes are serially measured even if eventually the patient does not undergo actual fertility preservation. Since there are very few prospective studies assessing gonadal function and fertility in survivors of childhood cancer, a longitudinal evaluation is imperative, particularly with all newer regimens which their gonadal effect remains to be elucidated. We also developed a parallel preclinical setting to study mechanisms of gonadotoxicity of anti-cancer treatments in a mouse model. Clinical dilemmas and questions are being translated into studying the effect in the animal setting, while themes and outcomes from the preclinical setting are being implemented into observational studies in the patient cohort (i.e. biomarkers for gonadal toxicity).

Due to the uncertainty over the risk of gonadotoxicity and infertility associated with different treatment protocols in children and young adults, it is mandatory to create a “risk assessment” model to guide the decision on referral to FP. The indication for FP in adolescents has become broader since FP options have become standard (sperm banking or oocyte cryopreservation) and not experimental. Our strategy is to discuss these options with all patients that are candidates for chemotherapy, not only highly gonadotoxic (i.e. ABVD protocol). For the prepubertal girls, risk assessment is performed by the treating oncologist. High risk patients are referred for FP, while intermediate risk are usually referred for discussion, and the actual decision on FP would be made by the joined professional team and the parents. Our results indicate that in chemotherapy-naïve patients the yield of isolating immature oocytes from the retrieved ovary and the success rate of IVM is significantly higher than in previously treated patients (even with less gonadotoxic regimens as anthracyclines). We therefore suggest a “risk assessment” process before starting any treatment. If the chances of protocol conversion into a high gonadotoxic regimen are high, the case should be discussed in a multidisciplinary setting to weigh whether the patient should be referred in advance for FP to enhance the yield of fertility preservation.

## Conclusion

With the advances of cancer treatments and the subsequent rise in survival, fertility has emerged as a highly significant quality-of-life issue for young cancer patients. Despite pediatric oncologists’ motivation to preserve fertility in pediatric cancer patients, barriers to referral for FP still persist. Multidisciplinary programs that encompass experts of all relevant fields, skilled laboratory resources and a facilitated path that eases the process and conveys it in the shortest time, appear to maximize the yield. We have observed a considerable higher referral rates compared with the previous experience before the launch of the program and higher satisfaction rate among the patients and their parents that consider the program as “an address” for fertility issues.

## Abbreviations

AMH, anti mullerian hormone; ASCO, American Society of Clinical Oncology; ASRM, American Society for Reproductive Medicine; BMT, bone marrow transplantation; FP, fertility preservation; IVM, in vitro maturation; OCP, ovarian cryopreservation
